# Interventional Techniques for the Management of Knee Osteoarthritis: A Literature Review

**DOI:** 10.7759/cureus.47133

**Published:** 2023-10-16

**Authors:** Kashif N Malik, Nathan Camp, Justin Chan, Matthew Ballard

**Affiliations:** 1 Physical Medicine and Rehabilitation, Casa Colina Hospital, Pomona, USA; 2 Pain Management, Western University of Health Sciences, Pomona, USA

**Keywords:** platelet-rich plasma therapy for joints, intra-articular platelet-rich plasma, intra-articular steroids, cryoneurolysis, visco-supplementation, dextrose prolotherapy, interventional pain medicine, osteoarthritis knee

## Abstract

Osteoarthritis of the knee is a prevalent condition that causes pain, discomfort, and disability that can severely impact the quality of life. This literature review aims to review the various interventional pain management techniques available to treat knee osteoarthritis. It analyzes the efficacy of various interventions such as intra-articular corticosteroids, prolotherapy, viscosupplementation, platelet-rich plasma, and genicular nerve blocks with radiofrequency ablation or cryoneurolysis. We searched databases for studies published in the past 20 years. A total of 37 articles were included. The literature supports the idea that a comprehensive treatment plan consisting of the various aforementioned techniques can provide relief for patients while delaying or avoiding joint replacement surgery.

## Introduction and background

Osteoarthritis of the knee is a leading cause of disability [1]. The current management is typically limited to the treatment of symptoms until the late stages of arthritis lead to knee replacement. There are a variety of tools to manage osteoarthritis including medications, physical therapy, procedures, and surgery. Intra-articular glucocorticoid injections are commonly used as a primary treatment for osteoarthritis of the knee, as suggested by an epidemiologic analysis of clinical practice guidelines for the non-arthroplasty treatment of osteoarthritis of the knee. Overall, 43.5% of all patients treated with any knee arthroplasty (total knee, unicompartmental, or patellofemoral arthroplasty) were administered intra-articular corticosteroid injections within five years of end-stage treatment, and 15.4% were administered viscosupplementation injections within five years of arthroplasty [2]. The literature reports that complications from these injections occur infrequently but can include joint infection, accelerated degradation of articular cartilage, and subchondral insufficiency fractures [3-5].

Recently emerging in the field are newer techniques such as genicular nerve radiofrequency ablations, prolotherapy, platelet-rich plasma (PRP), and cryoneurolysis.

A genicular nerve block is a minimally invasive procedure that is used to treat the pain associated with knee osteoarthritis. This procedure involves injecting a local anesthetic and a corticosteroid near the genicular nerves, which provide pain sensation to the knee, either under ultrasound or fluoroscopic guidance [6]. Only three of the four genicular nerves are blocked as blocking the inferolateral genicular nerve can cause a foot drop [7]. This procedure is well tolerated and can provide temporary relief of knee pain for approximately one to three months. If the patient gets relief from the nerve block, then radiofrequency ablation can be used to ablate these nerves to provide relief on average for six to 12 months [8].

Prolotherapy is a minimally invasive medical technique used to treat musculoskeletal conditions such as knee osteoarthritis [9]. This technique involves injecting a solution containing dextrose, an anesthetic, and normal saline into targeted areas of the body, such as ligaments, tendons, and joints, to trigger the body’s natural healing process [10]. The solution injected creates a local inflammatory response which causes tissue regeneration and collagen deposition via the recruitment of interleukins, growth factors, and stem cells [11]. These injections target ligaments and tendons around the knee joint. Prolotherapy strengthens weakened or damaged connective tissues, which, in turn, reduces pain and improves function [11].

PRP is an autologous biologic product that has gained significant attention as a novel treatment of osteoarthritis [12]. The mixture contains a higher concentration of platelets compared to normal blood plasma. The platelets contain a variety of growth factors, cytokines, and proteins that modulate cell functions and tissue repair processes [13]. A patient’s blood is drawn and then placed in a centrifuge to separate the different components, including red blood cells, white blood cells, plasma, and platelets. The plasma is then drawn off separately and used to inject into the target area.

PRP exerts its effects through the various bioactive molecules it contains. Normally, platelets release their contents upon activation, which occurs at sites of endothelial injury. The growth factors and cytokines in PRP initiate and modulate cellular processes involved in tissue regeneration and inflammation modulation such as platelet-derived growth factor (PDGF), fibroblast growth factor (FGF), and transforming growth factor-B (TGF-B) [14]. These factors attract stem cells and stimulate angiogenesis which facilitates tissue remodeling.

Cryoneurolysis applies extreme cold to peripheral nerve pathways via a closed system with a cannula and probe, typically with ultrasound guidance, to block nerve conduction [15]. A pressurized gas, usually nitrous oxide, flows down the tube into the probe which is a low-pressure zone. This causes volume expansion resulting in a significant drop in temperature, known as the Joule-Thomson effect [15]. No substance is injected into the patient, but the physical contact of the probe with surrounding tissues creates an ice ball. Goal temperatures are typically around -70°C causing axonotmesis or reversible damage to nearby nerves.

Wallerian degeneration then occurs around seven to 21 days post-treatment, which causes degeneration of the distal end of the axon in an anterograde fashion [16]. The axon will then regenerate 1-2 mm per day approximately. Thus, the duration of cryoneurolysis depends on several factors including nerve diameter, surrounding tissue temperature, and distance from the cryoneurolysis treatment to the terminal nerve ending [16]. In 2014, the Food and Drug Administration (FDA) approved a handheld device called the Iovera, which has made the delivery of cryotherapy safer and more convenient with removable nitrous oxide canisters, and smart probes [17]. The treatment typically takes about 30 minutes. This can be used for chronic knee osteoarthritis pain and is used before knee replacement surgery to reduce postoperative pain [18].

## Review

Methodology

In this literature review, the authors searched PubMed Central and Google Scholar using the following keywords: osteoarthritis; knee; intra-articular injections; platelet-rich plasma; prolotherapy management of knee osteoarthritis; corticosteroid knee osteoarthritis; viscosupplementation; genicular nerve block. A total of 75 articles were selected in the primary screening but only 37 met the inclusion criteria. All studies included were conducted between 2000 and 2020. The main findings of each study are illustrated in Tables 1-3.

**Table 1:** Summary of articles discussing intra-articular corticosteroids and viscosupplementation.

Article title	Authors	Journal of publication	Publication year	Summary
Comparison between intra articular Botulinum toxin type A, corticosteroid, and saline in knee osteoarthritis: a randomized controlled trial [1]	Mendes et al.	Clinical Rehabilitation	2019	This study compared the effectiveness of intra-articular injection with Botulinum toxin type A, triamcinolone hexacetonide, or saline in primary knee osteoarthritis. At four weeks, the triamcinolone group showed better results than the botulinum and normal saline group concluding that intra-articular injection with a steroid had a higher effectiveness than that with botulinum or normal saline. This was assessed over four weeks using the WOMAC scale, and ultrasound measurement of synovial hypertrophy
Long-term effectiveness of intra-articular injections on patient reported symptoms in persons with knee osteoarthritis [2]	Liu et al.	The Journal of Rheumatology	2018	Researchers examined the long-term effectiveness of corticosteroid or hyaluronic acid injections in relieving symptoms in patients with knee osteoarthritis. Among 412 participants receiving injections, 77.2% received corticosteroid injections and 22.8% received hyaluronic acid injections. Approximately 18.9% of patients had additional injections after the initial injection. Switching between injectates was common. Compared to the placebo group, participants who received a corticosteroid injection experienced an improvement in pain, stiffness, and physical functioning
Kinetics features changes before and after intra-articular hyaluronic acid injections in patients with knee osteoarthritis [3]	Tang et al.	Clinical Neurology and Neurosurgery	2015	25 subjects with bilateral symptomatic knee osteoarthritis and 15 healthy control subjects were recruited. Gait analyses were performed in both control and osteoarthritis groups before and after the completion of intra-articular hyaluronic acid injections at 1 week, 3 months, and 6 months. The visual analog pain scale and the Lequense index scores were both improved in the OA group after the hyaluronic acid injections
Viscosupplementation for knee osteoarthritis: systematic review and meta-analysis [4]	Pereira et al.	BMJ	2022	Researchers compiled 169 trials consisting of 21,163 randomized participants who received viscosupplementation with hyaluronic acid for knee osteoarthritis. They concluded that viscosupplementation leads to a small reduction in knee pain compared to placebo but also had an increased risk of adverse events compared to placebo. Their findings did not support the broad use of viscosupplementation for knee osteoarthritis
The evaluation of the effectiveness of intra-articular steroid, tenoxicam, and combined steroid–tenoxicam injections in the treatment of patients with knee osteoarthritis [5]	Yilmaz et al.	Clinical Rheumatology	2019	90 patients were randomly divided into three groups consisting of 30 patients each. Group 1, group 2, and group 3 were treated by intra-articular injection of tenoxicam, triamcinolone hexacetonide, and triamcinolone hexacetonide plus tenoxicam, respectively. The combined therapy produced a more effective result for a long period compared to monotherapy in reducing pain and improving functional recovery
Effect of intra-articular triamcinolone vs saline on knee cartilage volume and pain in patients with knee osteoarthritis: a randomized clinical trial [19]	McAlindon et al.	JAMA	2017	This study evaluated intra-articular triamcinolone vs normal saline for symptomatic knee osteoarthritis with ultrasonic features of synovitis in 140 patients. Intra-articular triamcinolone resulted in significantly greater cartilage volume loss compared to saline for a mean change in index compartment cartilage thickness of −0.21 mm in the steroid group versus −0.10 mm in the saline group. The researchers found no significant difference in pain in patients treated with steroids compared to saline
A comprehensive review of viscosupplementation in osteoarthritis of the knee [20]	Peck et al.	Orthopedic Reviews	2021	Researchers evaluated multiple studies comparing the different types of viscosupplementation. Some RCTs showed pain improvement with the viscosupplementation, while other RCTs reviewed by the researchers were inconclusive in terms of pain and functioning. It appeared the injectate was most effective when delivered in 2–4 weekly injections
Physical therapy versus glucocorticoid injection for osteoarthritis of the knee [21]	Deyle et al.	The New England Journal of Medicine	2020	156 knee osteoarthritis patients were assigned to either receive physical therapy only or a corticosteroid. Patients with who underwent physical therapy had less pain and functional disability at 1 year compared to patients who received an intra articular corticosteroid injection and no physical therapy
Intra-articular injections of platelet-rich plasma, hyaluronic acid or corticosteroids for knee osteoarthritis [22]	Huang et al.	Der Orthopäde	2019	120 patients were randomized to Intra-articular hyaluronic acid, corticosteroid or platelet-rich plasma for 3 weeks. There was a significant improvement in all scores (WOMAC, VAS) in each group compared to the pretreatment values. The clinical efficacy of PRP is comparable to that of the HA injectate. After 3 months, the long-term efficacy of PRP is superior to HA and corticosteroid
Viscosupplementation for osteoarthritis of the knee: a systematic review of the evidence [23]	Jevsevar et al.	Journal of Bone and Joint Surgery	2015	After analysis of multiple RCTs and review articles assessing the efficacy of hyaluronic acid, the primary authors concluded that the literature did not show clinically important differences in pain, stiffness, and activity level in patients that received hyaluronic acid versus placebo
Viscosupplementation in patients with severe osteoarthritis of the knee: six month follow-up of a randomized, double-blind clinical trial [24]	Campos et al.	International Orthopedics	2017	143 knee osteoarthritis patients were randomized into three groups receiving different intra-articular injections including triamcinolone, hyaluronic acid, or triamcinolone and hyaluronic acid. Outcomes were evaluated with the KSS and Lysholm score before and after treatment after one, three and six months. Viscosupplementation increased functional scores in patients with severe knee osteoarthritis; however, was not superior to the use of triamcinolone

**Table 2:** Summary of articles discussing prolotherapy and platelet-rich plasma.

Article title	Authors	Journal of publication	Publication year	Summary
Dextrose prolotherapy in knee osteoarthritis: a systematic review and meta-analysis [10]	Wee et al.	Journal of Clinical Orthopedic Trauma	2021	Eleven articles were selected consisting of 837 patients who had knee osteoarthritis. Prolotherapy for the knee was no different than platelet-rich plasma for the knee at 6 months for the pain scale. However, prolotherapy was found to be inferior compared to platelet-rich plasma at the 6-month mark for stiffness. Both procedures were well tolerated with minimal adverse effects
Comparing dextrose prolotherapy with other substances in knee osteoarthritis pain relief: a systematic review [7]	Cortez et al.	Clinics	2022	The aim of this study is to assess the effectiveness of dextrose-prolotherapy in alleviating pain in individuals with primary knee osteoarthritis, comparing it with alternative substances. The review focused on randomized clinical trials involving patients with primary knee osteoarthritis who received treatment with dextrose-prolotherapy or other substances for pain relief. The review findings indicate that individuals treated with dextrose-prolotherapy experienced improvements in pain levels from their baseline assessments, particularly when compared to saline injections. However, when compared to alternative substances, the results were inconclusive. While dextrose-prolotherapy stands as a viable treatment option on its own, it remains uncertain whether it is superior or inferior to alternative treatments. There is a pressing need for further research to generate more conclusive evidence in this field
Prolotherapy for osteoarthritis and tendinopathy: a descriptive review [25]	Rabago et al.	Rheumatology Report	2017	A systematic review, encompassing meta-analysis and randomized controlled trials, suggests that prolotherapy may offer symptomatic relief for mild-to-moderate knee osteoarthritis and overuse tendinopathy. Despite an incomplete understanding of its mechanism of action, which likely involves multiple factors, an increasing body of evidence indicates that prolotherapy may be a viable option for managing symptoms in carefully selected patients who have not responded to conventional therapies for knee osteoarthritis
The effects of injecting intra-articular platelet-rich plasma or prolotherapy on pain score and function in knee osteoarthritis [26]	Rahimzadeh et al.	Clinical Interventional Aging	2018	In this study, the researchers investigated the impact of platelet-rich plasma (PRP) injection and prolotherapy (PRL) on pain levels and knee joint function in osteoarthritis (OA) patients. The study included 42 knee OA patients who met specific criteria and participated in a randomized, double-blind clinical trial. Pain and knee function were assessed using the Western Ontario and McMaster Universities Osteoarthritis Index (WOMAC) both before and at various intervals after receiving PRP or PRL injections. The results showed a rapid improvement in WOMAC scores during the first and second months in both treatment groups, with a slight increase in the sixth month but still lower than the initial scores. Overall, the findings indicate that both PRP and PRL therapies can lead to significant improvements in the quality of life of knee OA patients shortly after treatment, with PRP showing superior effectiveness
Meta-analysis of clinical trials focusing on hypertonic dextrose prolotherapy (HDP) for knee osteoarthritis [11]	Wang et al.	Aging and Clinical Experimental Research	2022	This systematic review and meta-analysis investigated the effectiveness of hypertonic dextrose prolotherapy (HDP) injections in treating knee osteoarthritis (OA) by examining randomized controlled trials (RCTs) retrieved from MEDLINE, EMBASE, and Cochrane Library. Five studies involving a total of 319 patients were included showcasing that the HDP treatment, which involves injecting a concentrated dextrose solution, significantly improved knee OA outcomes at an average follow-up of 22.8 weeks. This improvement was seen in total WOMAC scores, pain levels, and knee function compared to control groups. No severe adverse events related to dextrose injections were reported in any of the included studies, indicating a reasonable safety profile for HDP. The study suggests that HDP holds promise as a treatment option for knee OA, and further research is needed to understand its mechanisms of action and long-term effects
The effectiveness of prolotherapy in treating knee osteoarthritis in adults: a systematic review [27]	Hassan et al.	British Med Bulletin	2017	To assess the efficacy of prolotherapy, the researchers conducted a literature review, Level 1 to Level 4, spanning databases to December 2016. Ten studies were scrutinized, revealing notable improvements in pain scores, functional capabilities, and range of motion for OA patients, both in the short and long term. Patient satisfaction rates reached a high of 82%. There is moderate evidence supporting the safety and effectiveness of prolotherapy in managing OA symptoms
Effect of intra-articular platelet-rich plasma vs placebo injection on pain and medial tibial cartilage volume in patients with knee osteoarthritis: the RESTORE randomized clinical trial [28]	Bennell et al.	JAMA	2021	This study aimed to assess the impact of platelet-rich plasma (PRP) injections on knee osteoarthritis (OA) symptoms and joint structure in patients with mild-to-moderate medial knee OA. The research involved a randomized, double-blind trial with 288 participants aged 50 years or older, conducted from August 2017 to July 2020 in Australia. The participants received either PRP or a saline placebo through intra-articular injections over 12 months. The study’s primary outcomes were changes in knee pain scores and medial tibial cartilage volume, both assessed at the 12-month mark. The results showed that PRP treatment did not lead to significant differences in knee pain or joint structure compared to the placebo. The findings do not support the use of PRP for managing knee OA symptoms and joint health
Platelet-rich plasma for the management of hip and knee osteoarthritis [12]	Bennell et al.	Rheumatology Report	2017	This narrative paper focuses on platelet-rich plasma (PRP) as a potential treatment for knee and hip osteoarthritis (OA) and primarily reviews evidence from randomized controlled trials (RCTs). Overall, the results indicate that PRP is a safe treatment option with the potential to offer short-term symptomatic relief for OA, particularly among younger patients with less severe disease
The effect of platelet-rich plasma on the intra-articular microenvironment in knee osteoarthritis [13]	Szwedowski et al.	International Journal of Molecular Science	2021	Knee osteoarthritis (KOA) poses a clinical challenge due to the limited natural healing capacity of cartilage lesions. Various treatments are available for KOA, ranging from oral non-steroidal anti-inflammatory drugs to physical therapy, braces, activity modifications, and surgical interventions. Intra-articular (IA) injections are commonly employed when non-operative treatments prove ineffective but surgery is not yet warranted. Recent studies indicate that IA injections may be as effective, if not more so, and safer than NSAIDs. Current research aims to enhance intra-articular homeostasis, with a focus on biologic adjuncts like platelet-rich plasma (PRP). PRP has the potential to influence the catabolic and inflammatory processes within the joint affected by KOA, potentially triggering regenerative responses and improving the metabolic functions of damaged structures. This review discusses inflammation and chondrogenesis mechanisms involved in cartilage repair and regeneration following PRP administration in in vitro and animal research. Additionally, it examines clinical trials assessing PRP’s effectiveness in altering OA biomarkers within the knee joint
Intra-articular platelet-rich plasma injection for knee osteoarthritis: a summary of meta-analyses [14]	Chen et al.	Journal of Orthopedic Surgery RES	2019	Based on short-term follow-up data (up to 1 year), intra-articular PRP injection appears to be more effective in relieving pain and improving function in KOA patients compared to HA and placebo injections. Moreover, there is no significant difference in the risk of adverse events between PRP and HA or placebo treatments
Platelet-rich plasma versus hyaluronic acid injections for the treatment of knee osteoarthritis: results at 5 years of a double-blind, randomized controlled trial [29]	Di Martino et al.	Am J Sports Med	2019	Platelet-rich plasma (PRP) injections have emerged as a potential non-surgical option for alleviating symptoms and delaying surgery in individuals with knee degeneration. This study aimed to compare the extended clinical outcomes of intra-articular injections of PRP and hyaluronic acid (HA) for treating knee degenerative conditions. In this randomized controlled trial, 192 patients with chronic symptomatic knee degeneration and osteoarthritis (Kellgren-Lawrence grade 0-3) were enrolled. They received three weekly intra-articular injections of either PRP or HA, with evaluations conducted before the injections and at 2, 6, 12, and 24 months, followed by a mean follow-up of 64.3 months. Assessment relied on the International Knee Documentation Committee (IKDC) subjective score as the primary outcome, alongside the EuroQol visual analog scale and Tegner scores. A total of 167 patients reached the final evaluation. The results demonstrated that both PRP and HA treatments effectively improved knee function and symptom relief over time. The IKDC subjective scores significantly improved for both groups and remained stable for up to 24 months. However, at the final evaluation, a significant reduction was observed in both groups, with the PRP group still maintaining significantly higher scores compared to the baseline. Comparative analysis revealed no substantial differences between the two treatments at any follow-up point, except for a lower rate of reintervention at 24 months in the PRP group (22.6% compared to 37.1% for HA)
The use of platelet-rich plasma in symptomatic knee osteoarthritis [30]	Southworth et al.	J Knee Surgery	2019	As the average life expectancy increases and obesity becomes more prevalent, osteoarthritis (OA) is becoming a growing financial and physical burden on the US population. OA develops as the body ages and experiences joint trauma, gradually wearing away the articular cartilage surfaces. Traditionally, treatment options have involved lifestyle changes, pain management, and corticosteroid injections, with joint replacement considered for those who have exhausted non-surgical approaches. More recently, treatments like hyaluronic acid, micronized dehydrated human amniotic/chorionic membrane tissue, and platelet-rich plasma (PRP) injections, have gained attention. PRP has demonstrated both anti-inflammatory effects, mediated by growth factors like transforming growth factor-β and insulin-like growth factor 1, and stimulatory effects on mesenchymal stem cells and fibroblasts. Several studies have suggested that PRP outperforms hyaluronic acid and corticosteroids in terms of improving patient-reported pain and functionality scores. This review assesses the existing literature on the use of PRP in treating symptomatic knee OA and offers recommendations for future research in this field
Platelet-rich plasma versus hyaluronic acid in the treatment of knee osteoarthritis: a meta-analysis [31]	Chen et al.	Medicine (Baltimore)	2020	This meta-analysis aims to address the ongoing debate regarding the effectiveness and safety of platelet-rich plasma (PRP) in comparison to hyaluronic acid (HA) for the clinical management of knee osteoarthritis. The goal is to establish an evidence-based medical approach for the conservative treatment of knee osteoarthritis while incorporating the most recent relevant research. The study also conducts a staged analysis to compare how PRP and HA therapy perform in different time periods. The meta-analysis included 14 randomized controlled trials (RCTs) encompassing 1350 patients. The results showed that the PRP group had higher scores in long-term VAS, IKDC, WOMAC-Pain, WOMAC-Stiffness, WOMAC-Physical Function, and WOMAC-Total scores at each evaluation point compared to the HA group. However, there were no significant differences between the two groups in the remaining indicators. In conclusion, PRP appears to offer clear advantages over HA in the conservative treatment of knee osteoarthritis. PRP treatment can lead to long-term pain reduction and improved knee joint function without introducing additional risks. Therefore, PRP can be a widely applicable option for conservative knee osteoarthritis treatment

**Table 3:** Summary of articles discussing genicular nerve blocks and cryoneurolysis.

Article title	Authors	Journal of publication	Publication year	Summary
Ultrasound-guided genicular nerve block for knee osteoarthritis: a double-blind, randomized controlled trial of local anesthetic alone or in combination with corticosteroid [32]	Kim et al.	Pain Physician	2018	A randomized, double-blinded study aiming to compare the analgesic effects of genicular nerve block (GNB) with lidocaine vs. lidocaine plus triamcinolone (TA). The results showed that the lidocaine plus TA group had significantly lower VAS scores at both 2 (p < 0.001) and 4 (p < 0.001) weeks after GNB. Clinical function was measured using the Oxford Knee Score (OKS) which showed the lidocaine plus TA group had significantly lower scores at 4 weeks compared to the lidocaine group; however, both groups returned to baseline functional levels by 8 weeks
Ultrasound-guided genicular nerve block versus physical therapy for chronic knee osteoarthritis: a prospective randomised study [33]	Güler et al.	Rheumatology International	2022	102 patients participated in a prospective randomized controlled trial comparing ultrasound-guided genicular nerve block (GNB) to physical therapy (PT) in patients with chronic knee osteoarthritis (OA). Results showed that VAS scores at baseline, 2 weeks, and 12 weeks were comparable between the two groups. Additionally, other outcome measures including the WOMAC-Total, WOMAC-Pain, and WOMAC-Physical were comparable. The WOMAC-Stiffness showed decreased scores in the GNB group at 2 and 12 weeks. Function was measured with a 6-minute walk test which showed considerable improvement in the GNB group compared to the PT group at 2 and 12 weeks
Genicular nerve block for pain management in patients with knee osteoarthritis: a randomized placebo-controlled trial [6]	Shanahan et al.	Arthritis & Rheumatology	2023	59 patients were enrolled in a 12-week parallel-group, placebo-controlled randomized trial of GNB for knee osteoarthritis. Patients in the active group (celestone chronodose plus bupivacaine) had significant improvement in VAS pain scores compared to placebo (normal saline) at 4, 8, and 12 weeks with a diminution of the effect over time. Total WOMAC scores were also significantly improved in the active group during that same time period
Genicular nerve radiofrequency ablation for painful knee arthritis: the why and the how [8]	Kidd et al.	JBJS Essential Surgical Techniques	2019	Genicular nerve radiofrequency ablation (GNRFA), including conventional, cooled, and pulsed techniques, is a new and innovative treatment for the management of knee osteoarthritis (OA). This can be indicated for patients with OA who have failed conservative treatments and surgical treatment or who are poor surgical candidates. GNRFA is a two-step procedure. First, patients are given a diagnostic block under fluoroscopy or ultrasound guidance around the superior lateral, superior medial, and inferior medial genicular nerve branches. If the patient reports a ≥50% reduction in baseline pain for a minimum of 24 hours following the injection, then the patient is a candidate for genicular ablation. General anesthesia is not required for GNRFA
Ultrasound-guided genicular nerve blockade with pharmacological agents for chronic knee osteoarthritis: a systematic review [9]	Tan et al.	Pain Physician	2022	A systemic review aimed to evaluate knee function following ultrasound-guided genicular nerve blocks using various pharmacological agents (corticosteroid, local anesthetic agents, and/or alcohol). Nine studies were included with a total of 280 patients. Follow-up intervals for pain and functional assessments were heterogeneous, ranging from one week to 6 months post-procedure. Sustained improvements in both pain and knee function were observed for up to 6 months regardless of the choice of pharmacological agents. Minimal adverse effects were reported
Is cooled radiofrequency genicular nerve block and ablation a viable option for the treatment of knee osteoarthritis? [34]	Carlone et al.	Arthroplasty Today	2021	A retrospective review evaluating demographic and psychosocial factors in 176 subjects with knee OA who underwent genicular nerve ablation, block, or both. Subjects who failed the initial block (31.8%) were significantly more likely to have psychological comorbidities, smoking history, and diabetes. These patient factors were not associated with second-stage ablation failures
A prospective randomized comparison of the efficacy of ultrasound- vs fluoroscopy-guided genicular nerve block for chronic knee osteoarthritis [35]	Kim et al.	Pain Physician	2019	A prospective randomized controlled study of 80 patients comparing ultrasound- vs fluoroscopy-guided genicular nerve blocks for chronic knee osteoarthritis. No differences in NRS-11 or WOMAC were observed between the two groups at baseline or during the follow-up period (1 and 3 months). Global Perceived Effect Scales (GPES) and complication rates were also similar between both groups
Effect of genicular nerve radiofrequency ablation for knee osteoarthritis: a retrospective chart review [36]	Fitzpatrick et al.	Wisconsin Medical Journal	2021	A retrospective study aimed at evaluating the efficacy of genicular nerve block and radiofrequency ablation in regard to pain and function in 18 patients with knee osteoarthritis. Both procedures were found to reduce pain in the post-procedure and follow-up settings. The Western Ontario and McMaster Universities Osteoarthritis Index correlate to reduced pain scores and may help identify appropriate candidates for these procedures
Preoperative cryoneurolysis for total knee arthroplasty: a case series [37]	Roth et al.	Journal of Perianesthesia Nursing	2023	A pilot project case series of 10 patients who underwent preoperative cryoneurolysis treatment prior to total knee arthroplasty (TKA). Results showed nine out of 10 patients had PACU pain scores of 0 out of 10 for 90 minutes postoperatively. Increased active range of motion was noted with reports of decreased pain scores during postoperative physical therapy sessions
Cryoneurolysis for the management of chronic pain in patients with knee osteoarthritis; a double-blinded randomized controlled sham trial [15]	Nygaard et al.	BMC Musculoskeletal Disorders	2021	A two-arm, parallel-group, RCT of 94 patients investigating if cryoneurolysis treatment is superior to sham treatment at decreasing pain intensity 2 weeks after intervention in patients with knee OA. This study is recently completed and is pending the final report. The obtained results will be made publicly available within 1 year after the end of the project
The evolution of cryoneurolysis for the treatment of shoulder, hip, and knee pain: where are we now and where will we go? A systematic review [17]	McMillan et al.	Surgical Technology International	2020	Cryoneurolysis has been described for decades across various specialties. Within the past few years, a growing movement of its application within orthopedics has provided pain relief solutions in both the non-surgical and surgical space. A review of the literature utilizing multiple medical search engines was performed to identify relevant orthopedic articles related to the treatment of joint pain with cryoneurolysis or cryoanalgesia. A review of the cryoneurolysis, indications, efficacy, and treatment gaps within the literature were identified to provide guidance for future research
Cryoneurolysis to treat the pain and symptoms of knee osteoarthritis: a multicenter, randomized, double-blind, sham-controlled trial [16]	Radnovich et al.	Osteoarthritis Cartilage	2017	A randomized, double-blinded, sham-controlled, multicenter trial of 180 patients evaluating the efficacy and safety/tolerability of cryoneurolysis for the reduction of pain and symptoms associated with knee osteoarthritis (OA). Cryoneurolysis of the infrapatellar branch of the saphenous nerve (IPBSN) resulted in statistically significant decreased knee pain and improved symptoms compared to sham treatment for up to 150 days and appeared safe and well tolerated
Percutaneous freezing of sensory nerves prior to total knee arthroplasty [18]	Dasa et al.	The Knee	2016	A retrospective chart review of 100 patients who underwent TKA to assess the value of perioperative cryoneurolysis. A significantly lower proportion of patients in the treatment group had a LOS of ≥ 2 days compared with the control group (6% vs. 67%, p < 0.0001) and required 45% less opioids during the first 12 weeks after surgery. There were significantly reduced symptoms and pain intensity reported at two and six-week follow-ups in the cryoneurolysis treatment group

There are a variety of non-surgical techniques available for the management of knee osteoarthritis. Knee osteoarthritis poses a significant health burden worldwide, prompting the exploration of diverse treatment modalities to alleviate pain, improve function, and potentially slow disease progression. This literature review critically evaluates and compares emerging therapeutic options for knee osteoarthritis including corticosteroids, viscosupplementation, prolotherapy, PRP, and genicular nerve blocks. Each intervention offers a distinct mechanism of action, clinical efficacy, safety profile, and future implications in the management of knee osteoarthritis.

Intra-articular corticosteroids are well-studied and have been utilized in the management of knee osteoarthritis for decades. For many patients, they are the first type of injection that they receive. Although the pain from osteoarthritis is mainly due to degeneration of the joint, there is often an inflammatory component that benefits from the anti-inflammatory effects of the steroid [19]. Many patients experience relief from these injections; however, their use is limited to approximately two to three times per year due to the cumulative chondrotoxicity and osteoporotic effects of the steroid. Additionally, there is some concern that corticosteroids may reduce pain that normally serves to protect the joint from overuse and thus allow patients to mobilize and promote further destruction of the joint [21].

Viscosupplementation is a relatively new technique that involves the injection of different formulations of hyaluronan into the joint. Hyaluronans are important biopolymers consisting of repeating disaccharide units of N-acetyl-d-glucosamine and d-glucuronic acid [20]. These molecules are a component of synovial fluid that provides lubrication to the joint and serves as a shock-absorbing layer between the components of the joint [23]. Recent literature also suggests these molecules can alter cell signaling pathways and decrease the expression of pro-inflammatory cytokines [11]. Commercially available formulations of hyaluronic acid are produced via extraction from animal tissue such as rooster combs. These formulations are injected into the joint space, with care to avoid injection into the fat pad, over a series of three to five injections a week apart [24]. These injections can be repeated approximately every six months.

Clinical studies evaluating prolotherapy’s efficacy have yielded mixed results. While some trials report reduced pain and improved function, others show limited benefit compared to placebo [7]. The variability in study designs, patient populations, and injection techniques may contribute to inconsistent outcomes. However, prolotherapy is usually very well tolerated with minimal side effects and can be an effective intervention in the appropriate group [25]. Prolotherapy can be repeated every four to six weeks and usually requires on average about three to five treatments for patients to see a benefit.

PRP has demonstrated promising results in multiple studies. Patients receiving PRP injections have reported reduced pain and improved function [28]. Some evidence suggests PRP might positively influence cartilage preservation and slow osteoarthritis progression, although long-term data are limited. These benefits likely stem from the growth factors present in the platelets, which then cause cellular remodeling, cell proliferation, and bone regeneration [12]. In most cases, patients report quality of life improvements following these injections and have higher rates of patient satisfaction compared to other interventions [14]. The benefit of PRP injections is that the injectate is an autologous product meaning it is obtained and derived from patients themselves. Theoretically, this means that PRP injections can be repeated more often and frequently than steroid injections and have a lower risk for side effects such as allergic reactions or disease transmission [13].

Genicular nerve blocks are typically used in patients with knee osteoarthritis unresponsive to other therapies. Some physicians may opt for a trial under ultrasound where the nerve is blocked with lidocaine or other local anesthetics [32]. If patients get relief from this trial, then many proceed to radiofrequency ablation of the genicular nerves performed under fluoroscopy. They can also be performed under ultrasound with similar efficacy, with the benefit of not being exposed to radiation [33]. Patients typically experience anywhere from three to 12 months of pain relief, with the average of patients experiencing six months of relief.

Cryoneurolysis is the use of cold temperatures to disrupt nerve conduction and can be used to address chronic knee pain related to osteoarthritis by targeting superficial sensory nerves [15]. Similar to genicular nerve radiofrequency ablation, it can be considered an alternative treatment for those patients who have chronic knee pain from osteoarthritis and conservative treatments have not been effective [16]. It can also be considered in those patients where surgery would be beneficial, but who are either not interested in surgical treatment or are poor candidates. With the advent of the Iovera, a handheld device developed in 2014, cryoneurolysis became more convenient, requiring less technical expertise to administer, and does not necessarily require imaging guidance [17]. It can be used before total knee arthroplasty and is found to be associated with reduced postoperative pain, reduced length of hospital stay following surgery, reduced opiate consumption, and increased range of knee motion during physical therapy [18]. Its effects last anywhere from three to six months.

There are constant innovations and research into the management and treatment of knee osteoarthritis. Future areas of research include combining multiple types of interventions to maximize the relief that patients can receive. For example, the combination of genicular nerve blocks with prolotherapy or PRP could yield synergistic effects, targeting both tissue repair and pain relief simultaneously. Other areas of research include optimizing patient selection for each intervention. Identifying specific patient characteristics that affect each intervention will allow physicians to select treatments that are uniquely tailored to each patient’s clinical condition and thus will enhance treatment outcomes and patient satisfaction. Long-term studies including randomized controlled trials continue to be needed to establish the sustained efficacy and safety of these therapies, particularly their impact on disease progression and patient outcomes over time.

## Conclusions

There are a variety of interventional techniques for the management of knee osteoarthritis. The previous mainstay of treatments included corticosteroid injections and viscosupplementation. However, there are emerging methods such as prolotherapy, PRP, and genicular nerve blocks that represent promising treatment options for knee osteoarthritis, each with unique mechanisms of action and clinical implications. PRP has shown consistent potential in reducing pain and improving function, while prolotherapy’s efficacy remains variable. Genicular nerve blocks either through heat radiofrequency ablation or cyroneurolysis offer effective pain relief without directly addressing the underlying disease. As research advances, a comprehensive understanding of these modalities’ benefits, limitations, and long-term effects will be crucial in guiding clinical decision-making and improving the quality of life for patients with knee osteoarthritis.
